# Oral Frailty and Social Frailty Among Older Japanese

**DOI:** 10.1111/ger.70056

**Published:** 2026-02-17

**Authors:** Isi Susanti, Hazem Abbas, Kenji Takeuchi, Jun Aida, Ken Osaka

**Affiliations:** ^1^ Department of Community Dentistry of Chulalongkorn University Bangkok Thailand; ^2^ Department of International and Community Oral Health Tohoku University Graduate School of Dentistry Sendai Japan; ^3^ Division of Statistics and Data Science, Liaison Center for Innovative Dentistry Tohoku University Graduate School of Dentistry Sendai Japan; ^4^ Department of Dental Public Health, Graduate School of Medical and Dental Sciences Institute of Science Tokyo Tokyo Japan

**Keywords:** aging, dentistry, frailty, public health, sociology

## Abstract

**Objective:**

To examine the association between oral frailty (OF) and social frailty (SF) among independent community‐dwelling older adults in Japan.

**Methods:**

A cross‐sectional study was conducted using data from the 2022 wave of the Japan Gerontological Evaluation Study (JAGES) including 19,319 functionally independent older adults aged ≥ 65 years. Oral frailty was used as an exposure variable in four ways: (1) a categorical variable (robust = 0, pre‐frail = 1, frail ≥ 2), (2) count score (sum of 5 OF components), (3) individual OF components and (4) a combined variable for the four oral functions. SF was measured using the 5‐item index (SF‐5; robust = 0, pre‐frail = 1, frail ≥ 2). Missing data were handled via multiple imputation. Ordered logistic regression models were used after adjusting for sociodemographic and health‐related covariates.

**Results:**

Participants were 48.4% males and aged 65–104 years. SF prevalence was 28.3% (*n* = 5466). In the adjusted models, oral frail individuals had higher odds of being socially frail (OR 1.35; 95% CI 1.26–1.45) than the robust group and the highest oral frailty score (score 5) showed the highest odds (OR 2.53; 95% CI 1.50–4.27) for SF. In addition, among OF components, **dry mouth** had the strongest association (OR 1.29; 95% CI 1.20–1.38), and those with **four diminished oral functions** were more likely to be socially frail (OR 2.42; 95% CI 1.53–3.82). Overall, a clear gradient‐like pattern was observed with more oral frailty linked to higher odds of social frailty.

**Conclusion:**

OF and its components were associated with SF among older adults in Japan. These findings might suggest the need for integrated screening and preventive strategies to mitigate social vulnerability linked to oral functional decline.

## 1 Introduction

Frailty has been increasingly recognised as a **multifaceted construct** encompassing not only physical but also psychological and social dimensions [1]. These aspects are closely interrelated and can collectively contribute to adverse health outcomes in later life [2, 3]. As global populations continue to age, maintaining functional capacity and quality of life among older adults has become a public health priority [4, 5, 6]. Although much of the existing literature has traditionally focused on **physical frailty**, growing evidence highlights that the **social dimensions of aging** play an equally critical role in shaping health trajectories [7, 8].

One important yet often underrecognised concept is **social frailty (SF)** [9]. While there is no universally accepted definition [9], SF is commonly described as a continuum of risk related to the loss of social, general or economic resources and activities that fulfil basic social needs [10]. SF reflects increased vulnerability to social disconnection, loneliness and reduced social participation [11]. Globally, an estimated **20% of older adults** experience some degree of social frailty [12, 13], which has been associated with an increased risk of depression, cognitive impairment, decreased physical activity and mortality [14, 15, 16, 17].

In **Japan**, where the proportion of adults aged 65 years and older reached **29.3% in 2025**, the highest in the world [18], the prevalence of social frailty among community‐dwelling older adults has been reported to range between **10.2% and 11.1%** [17, 19]. This figure underscores the need to address social frailty in aging societies. Given the growing burden of social frailty, identifying **modifiable risk factors** is crucial for prevention and intervention.

One potential modifiable risk factor is **oral frailty**, which refers to age‐related declines in multiple oral functions (number of teeth, oral function and oral hygiene) [20]. Evaluating OF as a composite measure is important, as it captures a broader picture of oral functional decline than any single indicator [20]. Studies have associated OF with systemic outcomes such as sarcopenia and increased mortality risk [21, 22]. Prior research hypothesised that oral frailty may impair eating, speaking and self‐esteem, leading to reduced self‐confidence and potentially accelerating social withdrawal and consequently accelerating the onset of social frailty [22, 23, 24, 25].

Despite the growing recognition of both SF and OF as components of geriatric syndrome [7, 26], **the empirical evidence linking these two domains remains scarce**. Most existing studies have focused on the associations between oral health indicators (e.g., number of teeth) and outcomes such as social isolation or loneliness [22]. However, it is important to distinguish the difference between social isolation and SF. Social isolation is an objective state characterised by a reduced quantity or frequency of social contacts and represents only one aspect of social vulnerability [27], while SF is a broader, multidimensional concept that includes limited social interactions, financial hardship and lack of social support, making it a vulnerable state characterised by deteriorating social engagement [9]. Previously, only one study conducted in Japan investigated the reverse association (i.e., social frailty to oral frailty), suggesting that a reduction in social frailty might contribute to a decline in oral health [28].

Addressing this gap in the research is essential to better understand the social implications of oral functional decline and to provide early preventive interventions. Accordingly, this study aimed to assess the association between oral frailty and social frailty among independent community‐dwelling older adults in Japan.

## 2 Material and Methods

This cross‐sectional study utilised the data from the 2022 wave of the Japan Gerontological Evaluation Study (JAGES), a large‐scale, ongoing survey investigating the health, social and behavioural characteristics of community‐dwelling Japanese adults aged 65 years and older [29]. JAGES is a collaborative effort between academic institutions and local municipalities across Japan. Depending on municipal policies, population size and available budget, sampling methods varied, with some municipalities conducting complete surveys of all residents aged 65 and over and others using simple random sampling or a combination of both approaches [29]. For the present analysis, the target population included functionally independent older adults aged 65 and above, and individuals requiring assistance with daily activities were excluded.

### 2.1 Exposure *Variable*

The OF was determined using the OF‐5 index that included the following 5 items (fewer number of teeth, chewing, speaking and swallowing difficulties, and dry mouth experience) [30]. A self‐reported number of teeth question, ‘How many natural teeth do you presently have? Natural teeth include replanted, capped and wisdom teeth; there are a total of 32 permanent teeth’, was utilised and dichotomised into (less than 20 and 20 or more teeth groups). Participants with < 20 teeth were categorised as the fewer teeth group and were scored with one point. Four binary subjective self‐reported questions were used as follows: “Compared to six months ago, do you have difficulty eating hard foods?”, “Do you sometimes choke on tea or soup?”, ‘Are you concerned about thirst?’ and ‘Within the past 6 months, have you experienced difficulty in speaking?’ Participants who responded ‘yes’ were categorised as those with a decline in oral functions and were scored with one point for each ‘yes’ answer. It is worth mentioning that the original OF‐5 item on ‘low articulatory oral motor skills’ *clinically measures oral diadochokinetic in producing the ‘ta’ sound, however* in this study, *it* was adapted to the self‐reported question: ‘*Within the past 6 months, have you experienced difficulty in speaking?’* as JAGES data were naturally self‐reported and did not include clinical measures. This self‐reported question introduces a recall period and serves as a practical self‐reported proxy to capture early articulatory decline in a large‐scale survey context, as it reflects the same underlying construct (i.e., problems in speaking) [22, 30, 31]. During the analyses, oral frailty was used as exposure variable in four ways: (1) categorical variable: individuals who did not have any unfavourable answers (scored 0) and were categorised as the oral robust group, while those who had one unfavourable answer (scored 1) were classified as the pre‐oral frail group, and those who had two or more unfavourable answers (score ≥ 2) were classified as the oral frail group [30], (2) count score (sum of five components: number of teeth, chewing difficulty, swallowing difficulty, dry mouth, speaking difficulty), (3) individual oral frailty components with the number of teeth component analysed using three different binary categorisations (< 20 vs. ≥ 20, edentulous vs. non‐edentulous and < 5 vs. ≥ 5 teeth) and (4) a combined variable for the four oral functions.

### 2.2 Outcome Variable

The 5‐item social Frailty index (SF‐5), which was recognised as the most commonly utilised social frailty index was used in this study [19, 32]. It included five binary questions as follows: ‘Do you go out less frequently compared with last year?’ (Yes), ‘Do you sometimes visit your friends?’ (no), ‘Do you feel you are helpful to others?’ (no), ‘Which of the following best describes your family composition?’ (I live alone), and ‘What kind of friends or acquaintances do you meet frequently?’ (none). Individuals with no social withdrawal answers were classified as socially robust (score = 0); those with one answer indicating social withdrawal were classified as socially pre‐frail (score = 1), and those with two or more social withdrawal answers were classified as socially frail (score ≥ 2) [19]. It is worth mentioning that the original SF‐5 item ‘Do you talk with someone every day?’ was adapted in our study to the question ‘What kind of friends or acquaintances do you meet frequently?’ with its eight available answers: ‘1. Neighbours/people in the same area, 2. Childhood friends, 3. Friends from school, 4. Colleagues or former colleagues at work, 5. Friends with the same hobbies or interests, 6. Friends from volunteer activities, 7. Other and 8. None’. The eight answers were dichotomised into ‘meet frequently with *friends or acquaintances (*answers 1 to 7*)’* and ‘did not meet frequently with *friends or acquaintances (*answer 8)’ to capture similar information that could have been obtained from the original SF‐5 item (i.e., talking to someone every day). This adaptation should be considered when interpreting the findings while recognising its conceptual alignment with the original SF‐5 framework.

### 2.3 Covariates

Based on previous research examining resembling exposure and outcome [10, 22], the following variables were included as covariates in the analysis: age, sex, educational attainment, household income, residential area, self‐reported general health status and depression status [10]. Age was defined as the participant's age in years at the time of the survey and was treated as a continuous variable in the main analyses; for descriptive purposes, age was also categorised into predefined age groups (65–69 years, 70–74 years, 75–79 years, 80–84 years and ≥ 85 years). Sex was operationalised as a binary variable (male or female) as recorded in the original survey dataset. Educational attainment was categorised into four categories: less than 6 years, 6 to 9 years, 10 to 12 years and 13 years or more. Income was measured using equivalised household income to account for differences in household size and composition, providing a more accurate reflection of the respondent's economic resources [33]. Health status was categorised as excellent, good, fair, or poor based on self‐report. The residential area was categorised as urban, suburban or rural. Meanwhile, depressive symptoms were measured using the geriatric depression scale (GDS‐15) [34]. The cut‐off for the GDS score was interpreted as: 0–4 normal, 5–8 mild depression, 9–11 moderate depression and 12–15 severe depression [35].

### 2.4 Statistical Analysis

Descriptive statistics were employed to explore the characteristics of the study participants. Cross‐tabulation and chi‐squared test were used to compare differences in participants' characteristics between groups. Crude and adjusted ordered logistic regression models were applied to assess the association between oral frailty and social frailty using four different ways of assessing oral frailty to identify which component was the most strongly associated with social frailty. Missing data were addressed through multiple imputation (MI). Missing values were assumed to be missing at random (MAR). Imputation was applied to all variables used in this study except for age, sex and residential area, as these variables were the identifying variables of the participants in JAGES and are provided as complete by the JAGES management office; respondents missing these variables are deemed ineligible for participation. Multiple imputation by chained equations was conducted, generating 20 imputed data sets. Ordered logistic regression analyses were performed within the MI framework using MI‐enabled procedures, which fit models across all imputed datasets and automatically pooled parameter estimates and standard errors according to Rubin's rules. A sensitivity analysis using complete case data without imputation was conducted to validate the robustness of the main findings. All statistical analyses were performed using Stata/SE 18 (StataCorp LP). The significance level was set at α = 0.05. The study adhered to the STROBE guidelines for reporting cross‐sectional studies.

## 3 Results

A total of 19,319 participants aged 65–104 years were included in the analysis (mean age of 74.8 years, SD = 8.5 years), OF prevalence was 32.6% (*n* = 6307) and SF prevalence was 28.3% (*n* = 5466). Table 1 showed the characteristics of study participants by social frailty after multiple imputation. In summary, social frailty was more common among older participants, men, those with lower levels of income and education, poorer self‐rated health, residence in urban areas and higher depressive symptoms. The characteristics of the original unimputed data set were available in Supplementary Table S1.

**TABLE 1:** Characteristics of the study participants by social frailty after multiple imputation (*N* = 19,319).

Variable	Total		Social frailty		
	N	%	Robust	Social pre‐frail	Social frail
			N (%)	N (%)	N (%)
**Total**	**19,319**	**100**	**6673 (34.5)**	**7180 (37.2)**	**5466 (28.3)**
**Oral frailty category**					
Robust	7032	36.4	2799 (39.8)	2680 (38.1)	1553 (22.1)
Pre‐oral frail	5980	31	2086 (34.9)	2301 (38.5)	1593 (26.6)
Oral frailty	6307	32.6	1788 (28.3)	2199 (34.9)	2320 (36.8)
**Sex**					
Male	9356	48.4	2971 (31.76)	3583 (38.3)	2802 (29.9)
Female	9963	51.6	3702 (37.16)	3597 (36.1)	2664 (26.7)
**Age**					
65–69 years	4492	23.2	1568 (34.9)	1749 (38.9)	1175 (26.2)
70–74 years	5824	30.2	2143 (36.8)	2185 (37.5)	1496 (25.7)
75–79 years	4462	23.1	1635 (36.6)	1621 (36.3)	1206 (27.1)
80–84 years	3046	15.8	968 (31.8)	1104 (36.2)	974 (32)
≥ 85 years	1495	7.7	359 (24)	521 (34.9)	615 (41.1)
**Education**					
Less than 6 years	71	0.4	13 (18.3)	26 (36.6)	32 (45.1)
6 to 9 years	3832	19.8	1239 (32.3)	1327 (34.6)	1266 (33.1)
10 to 12 years	8621	44.6	3055 (35.4)	3155 (36.6)	2411 (28)
13 years or more	6795	35.2	2366 (34.8)	2672 (39.3)	1757 (25.9)
**Equivalised income**					
< 2,000,000 Yen/Year	9389	48.6	2874 (30.6)	3340 (35.6)	3175 (33.8)
2,000,000–2,999,999 Yen/Year	4632	24	1624 (35.1)	1778 (38.4)	1230 (26.5)
3,000,000–3,999,999 Yen/Year	2946	15.2	1188 (40.3)	1158 (39.3)	600 (20.4)
≥ 4,000,000 Yen/Year	2352	12.2	987 (42)	904 (38.4)	461 (19.6)
**Resident area**					
Urban	8818	45.6	2576 (29.2)	3425 (38.8)	2817 (32)
Suburban	2873	14.9	1017 (35.4)	1045 (36.4)	811 (28.2)
Rural area	7628	39.5	3080 (40.4)	2710 (35.5)	1838 (24.1)
**Health status**					
Excellent	2638	13.6	1198 (45.4)	991 (37.6)	449 (17)
Good	14,321	74.1	5047 (35.2)	5422 (37.9)	3852 (26.9)
Fair	2176	11.3	403 (18.5)	713 (32.8)	1060 (48.7)
Poor	184	1	25 (13.6)	54 (29.3)	105 (57.1)
**Depression status**					
Normal	14,774	76.5	5981 (40.5)	5726 (38.8)	3067 (20.7)
Mild Depression	3239	16.8	594 (18.3)	1129 (34.9)	1516 (46.8)
Moderate Depression	949	4.9	84 (8.8)	259 (27.3)	606 (63.9)
Severe Depression	357	1.8	14 (3.9)	66 (18.5)	277 (77.6)

*Note:* ‘All *p*‐values for chi‐squared were < 0.05’.

Table 2 showed the summary of the association between each oral frailty exposure and social frailty after multiple imputation. In all analyses, including both the crude and adjusted models, a clear gradient‐like pattern was observed (i.e., a higher oral frailty score was associated with higher odds of social frailty), and this pattern remained consistent across all oral frailty variables' analyses. Specifically, participants in the oral frail group had 35% higher odds of being socially frail than those in the oral robust group (OR = 1.35, 95% CI: 1.26–1.45), and participants with the highest **oral frailty score (score 5)** showed the strongest association (OR = 2.53; 95% CI 1.50–4.27) with SF. Among oral frailty components, **dry mouth** had the highest odds of social frailty (OR 1.29; 95% CI 1.20–1.38). Interestingly, this study found that dry mouth, chewing and swallowing difficulty had higher odds of being socially frail rather than having fewer than 20 teeth. However, when alternative numbers of teeth categorisations (edentulous vs. non‐edentulous and < 5 vs. ≥ 5 teeth) were applied, the odds ratios were slightly higher than the main number of teeth cut‐off (minimum functional dentition of 20 teeth for older adults) (Supplementary Table S4). Moreover, participants with **four diminished oral functions** had markedly higher odds of social frailty (OR 2.42; 95% CI 1.53–3.82) than those without diminished oral functions.

**TABLE 2:** The summary of the association between each oral frailty related exposure and social frailty after multiple imputation (*N* = 19,319)^a^.

Variables	Social frailty					
	Crude model			Adjusted model		
	OR	95%	CI	OR	95%	CI
**Oral frailty category**						
Robust (ref)	1	.	.	1	.	.
Preoral frail	1.24	1.16	1.33	1.13	1.05	1.21
Oral frailty	1.84	1.72	1.96	1.35	1.26	1.45
**Oral frailty score**						
0	1			1		
1	1.25	1.17	1.33	1.13	1.06	1.21
2	1.61	1.49	1.73	1.29	1.19	1.40
3	2.03	1.84	2.25	1.39	1.25	1.55
4	2.68	2.26	3.17	1.53	1.28	1.83
5	4.74	2.99	7.51	2.53	1.50	4.27
**Number of teeth**						
≥ 20	1			1		
< 20	1.21	1.15	1.28	1.09	1.02	1.16
**Chewing difficulty**						
No	1			1		
Yes	1.62	1.53	1.72	1.25	1.17	1.34
**Swallowing difficulty**						
No	1			1		
Yes	1.46	1.36	1.56	1.20	1.12	1.28
**Dry mouth**						
No	1			1		
Yes	1.69	1.58	1.81	1.29	1.20	1.38
**Speaking difficulty**						
No	1					
Yes	1.40	1.21	1.61	1.04	0.90	1.20
**Oral functions (OF)**						
0 diminished OF	1			1		
1 diminished OF	1.38	1.30	1.47	1.16	1.09	1.24
2 diminished OF	1.88	1.73	2.04	1.37	1.26	1.49
3 diminished OF	2.46	2.15	2.81	1.47	1.28	1.68
4 diminished OF	4.40	2.93	6.60	2.42	1.53	3.82

*Note:* Adjusted for: age, sex, education level, resident area, equivalised income, health status and depression status.

Abbreviations: CI, confidence interval; OR, odds ratio.

^a^ Each oral frailty related exposure was included in a separate model twice (a crude and an adjusted model), and the results were summarised in the above table.

Meanwhile, for sensitivity analyses examining the complete case analysis scenario, the direction and magnitude of associations between oral frailty and social frailty remained consistent with the main findings (Supplementary Table S2). In addition, the odds ratios for covariates of the model where the categorical OF exposure was examined were reported in Supplementary Table S3.

## 4 Discussion

This study examined the association between oral frailty and social frailty among 19,319 older adults in Japan. The findings demonstrated a clear gradient‐like pattern: individuals who had a higher oral frailty score had progressively higher odds of being socially frail. This pattern was observed in all analyses, showing the robustness of the association. Dry mouth, as an individual component of oral frailty, showed the strongest association with social frailty. These findings highlight that both the cumulative and component‐specific aspects of oral frailty were associated with social frailty among older adults in Japan.

This study had several strengths. First, to the best of our knowledge, it was the first study to examine the association between oral and social frailty among older adults. Second, the use of a relatively large sample size from the Japan Gerontological Evaluation Study (JAGES) enhanced the statistical power and overall reliability of the findings. Third, multiple imputation was utilised to address missing data, enhancing the validity and robustness of the findings. Fourth, the analytical models accounted for a broad range of sociodemographic and health covariates, reducing potential confounding. Fifth, the component‐level analysis provided a more nuanced understanding of which oral frailty item was most strongly linked to social frailty, offering valuable insights for potential interventions.

However, several limitations should be considered when interpreting the findings. Most notably, the cross‐sectional design limits causal interpretation, and without a clear time‐temporal sequence, it remains uncertain how oral frailty contributes to the development of social frailty. Also, including functionally independent older adults and excluding those requiring assistance with daily activities may have led to underestimation of the findings; however, this study deliberately focused on independent community‐dwelling older adults, as its aim was to assess the association between oral frailty and social frailty in a functionally independent population, rather than among older adults with functional limitations. Additionally, an item of the OF‐5 index (speaking difficulty) and an item of the SF‐5 index (daily conversation with someone) were adapted from similar questions in JAGES with different wording. These adaptations may affect the comparability of findings with other studies using the standard measures. Furthermore, the use of self‐reported measures for oral health status may introduce recall or misclassification bias; however, such measures are widely recognised as valid and practical in large‐scale epidemiological research [36, 37]. Furthermore, although the dataset used was relatively large, the sample was not nationally representative, which might have restricted the generalisability of the findings to the broader population of older adults in Japan. Lastly, it is worth noting that the average number of natural teeth among older adults in Japan is higher than in many other countries [38, 39]; this limited the generalisability of the findings to other countries as well.

The findings of this study were aligned with the previous literature showing that the **number of teeth**, chewing and swallowing difficulties and dry mouth, which were included in the OF‐5 index, were associated with decreased social engagement, increased social isolation and increased perceived loneliness, which are similar outcomes to social frailty [40, 41, 42]. A prior study also found a direct association between oral frailty using a similar OF‐5 index and decreased social engagement, supporting the notion that multidimensional oral function decline contributes to social vulnerability [22]. In addition, the findings of this study, if added to a previous study investigating the reverse association (i.e., social frailty to oral frailty) [28], suggest a bidirectional association between oral frailty and social frailty.

Interestingly, among oral frailty components, the number of teeth showed a weaker association than dry mouth, chewing and swallowing difficulty. This might be explained by dental prosthesis use. In Japan, many older adults use well‐fitted dentures and have relatively good structural access to dental care, which can help maintain masticatory function and appearance even with fewer natural teeth [43, 44, 45]. This interaction between the number of teeth and dental prosthesis use might explain the weaker association observed for the number of teeth than for dry mouth. Additionally, the cut‐off points for dichotomising the number of teeth influenced the magnitude of observed association, where lower cut‐offs showed higher magnitude (Supplementary Table S4) [46].

Potential mechanisms to explain the association between OF and SF might operate through decreased diet diversity or shifting to a soft diet due to tooth loss and masticatory difficulties, which may contribute to sarcopenia and decreased physical activity [22, 28, 47]. Swallowing difficulties can further worsen this issue by causing fear or anxiety during meals, discouraging eating in social settings and reinforcing isolation [42]. Additionally, dry mouth and the experience of speaking difficulty could hinder communication and thus lower self‐esteem, which consequently further impairs social interaction [40, 48, 49]. Thus, a decline in the number of teeth and oral functions might serve as a modifiable risk factor in the cascade leading to social frailty [15, 21, 50, 51].

Oral health tends to be neglected within universal health coverage [52, 53]. The high prevalence of social frailty among individuals with oral frailty suggests the need for enhanced oral health care for the older population. Incorporating routine screening for oral health into community‐based health initiatives may facilitate the timely identification of older adults at risk and support the development of targeted interventions [21, 54].

Future research examining the longitudinal relationship between OF and SF is needed to clarify the temporal sequence of this relationship, in addition to examining the different pathways and the causal relationship between oral frailty and social frailty among the aging population.

## 5 Conclusion

The experience of oral frailty and its components were associated with higher social frailty among older Japanese adults in this cross‐sectional study. Longitudinal studies are needed to explore the temporal relationship and causal pathways between OF and SF.

## Author Contributions

Isi Susanti contributed to the conceptualisation, study design, data acquisition, data analysis, interpretation of the findings and drafting of the manuscript. Hazem Abbas contributed to the conceptualisation, study design, data acquisition, data analysis, interpretation of the findings and manuscript development. Kenji Takeuchi contributed to the study design, data analysis, interpretation of the findings and manuscript development. Jun Aida and Ken Osaka contributed to data acquisition. All authors critically reviewed and revised the manuscript, approved the final version, and agreed to be accountable for all aspects of the work, ensuring its integrity and accuracy.

## Funding

This study received no specific funding; however, it used data from JAGES (the Japan Gerontological Evaluation Study). The JAGES was supported by Grant‐in‐Aid for Scientific Research (19K02200, 20H00557, 20H03954, 20K02176, 20K10540, 20K13721, 20K19534, 21H00792, 21H03196, 21K02001, 21K10323, 21K11108, 21K17302, 21K17308, 21K17322, 22H00934, 22H03299, 22J00662, 22J01409, 22K01434, 22K04450, 22K10564, 22K11101, 22K13558, 22K17265, 22K17364, 22K17409, 23K16320, 23H00449, 23H03117, 23K19793, 23K21500, 23K24557) from JSPS (Japan Society for the Promotion of Science), Health Labour Sciences Research Grants (19FA1012, 19FA2001, 21FA1012,22FA2001, 22FA1010, 22FG2001, 24FA1020), the Research Funding for Longevity Sciences from National Center for Geriatrics and Gerontology (21‐20), Research Institute of Science and Technology for Society (JPMJOP1831) from the Japan Science and Technology (JST), a grant from Japan Health Promotion & Fitness Foundation, contribution by Department of Active Ageing, Niigata University Graduate School of Medical and Dental Sciences (donated by Tokamachi city, Niigata), TMDU priority research areas grant and National Research Institute for Earth Science and Disaster Resilience. The views and opinions expressed in this article are those of the authors and do not necessarily reflect the official policy or position of the respective funding organisations.

## Ethics Statement

This study was approved by the Ethics Committee on the Research of Human Subjects of Chiba University (approval number: 2493) and the National Center for Geriatrics and Gerontology, Japan (approval number: 992‐3). Participants were notified that participation was voluntary, and returning an answered survey was considered consent to participate in the JAGES study.

## Conflicts of Interest

The authors declare no conflicts of interest.

## Supporting information


**Tables S1‐S4:** ger70056‐sup‐0001‐TablesS1‐S4.docx.

## Data Availability

The data from the Japan Gerontological Evaluation Study (JAGES) are available upon requests submitted to the JAGES data management office. Applications for data usage are accepted online from the JAGES website. https://www.jages.net/.
