# Supplementary material for: Oral Frailty and Social Frailty Among Older Japanese
Source: Gerodontology. 2026 Feb 17;43(2):252–60. doi: 10.1111/ger.70056 (PMC13140007; doi:10.1111/ger.70056)
Supplement: Supplementary file 1 — Tables S1‐S4: ger70056‐sup‐0001‐TablesS1‐S4.docx. [file GER-43-252-s001.docx]

**Supplementary Table 1. Characteristics of the study participants’ social frailty without multiple imputation (N = 19,319).**

|  |  |  | **Oral Frailty** | | |  |
| --- | --- | --- | --- | --- | --- | --- |
| **Variable** |  |  | **Robust** | **Socially**  **Prefrail** | **Socially Frail** | **Missing** |
|  | **N** | **%** | **N (%)** | **N (%)** | **N (%)** | **N (%)** |
| **Total** | **19,319** | **100** | **6,209 (32.1)** | **6,720 (34.8)** | **5,037 (26.1)** | **1,353 (7%)** |
| **Oral Frailty category** |  |  |  |  |  |  |
| Robust | 6,863 | 35.5 | 2,592 (37.8) | 2,494 (36.3) | 1,432 (20.9) | 345 (5) |
| Pre-oral frail | 5,844 | 30.2 | 1,921 (32.9) | 2,127 (36.4) | 1,460 (25) | 336 (5.7) |
| Oral Frailty | 6,147 | 31.8 | 1,637 (26.6) | 2,028 (33) | 2,083 (33.9) | 399 (6.5) |
| Missing | 465 | 2.4 | 59 (12.7) | 71 (15.3) | 62 (13.3) | 273 (58.7) |
| **Sex** |  |  |  |  |  |  |
| Male | 9,356 | 48.4 | 2,791 (29.8) | 3,383 (36.2) | 2,573 (27.5) | 609 (6.5) |
| Female | 9,963 | 51.6 | 3,418 (34.3) | 3,337 (33.5) | 2,464 (24.7) | 744 (7.5) |
| **Age** |  |  |  |  |  |  |
| 65-69 years | 4,492 | 23.2 | 1,468 (32.7) | 1,679 (37.4) | 1,116 (24.8) | 229 (5.1) |
| 70-74 years | 5,824 | 30.1 | 2,016 (34.6) | 2,033 (35) | 1,399 (24) | 376 (6.5) |
| 75-79 years | 4,462 | 23.1 | 1,515 (34) | 1,517 (34) | 1,113 (25) | 317 (7.1) |
| 80-84 years | 3,046 | 15.8 | 894 (29.3) | 1,018 (33.4) | 868 (28.5) | 266 (8.7) |
| >85 years | 1,495 | 7.7 | 316 (21.1) | 473 (31.6) | 541 (36.2) | 165 (11.4) |
| **Education** |  |  |  |  |  |  |
| Less than 6 years | 70 | 0.4 | 12 (17.1) | 24 (32.3) | 26 (37.1) | 8 (11.4) |
| 6 to 9 years | 3,768 | 19.5 | 1,109 (29.4) | 1,193 (31.7) | 1,120 (29.7) | 346 (9.2) |
| 10 to 12 years | 8,473 | 43.8 | 2,830 (33.4) | 2,890 (34.1) | 2,890 (275.8) | 568 (6.7) |
| 13 years or more | 6,688 | 34.6 | 2,183 (32.6) | 2,504 (37.4) | 1,611 (24.1) | 390 (5.8) |
| Missing | 320 | 1.7 | 75 (23.4) | 109 (34.1) | 95 (29.7) | 41 (12.8) |
| **Equivalized income** |  |  |  |  |  |  |
| <2,000,000 Yen/Year | 8,179 | 42.3 | 2,321 (28.4) | 2,721 (33.3) | 2,551 (31.2) | 586 (7.2) |
| 2,000,000 - 2,999,999 Yen/Year | 4,137 | 21.4 | 1,366 (33) | 1,517 (36.7) | 1,029 (24.9) | 225 (5.4) |
| 3,000,000 - 3,999,999 Yen/Year | 2,662 | 13.8 | 1,008 (37.9) | 1,011 (38) | 512 (19.2) | 131 (4.9) |
| > 4,000,000 Yen/Year (ref) | 2,138 | 11.4 | 842 (39.4) | 784 (36.7) | 392 (18.3) | 120 (5.6) |
| Missing | 2,203 | 10.6 | 672 (30.5) | 687 (31.2) | 553 (25.1) | 291 (13.2) |
| **Resident Area** |  |  |  |  |  |  |
| Urban | 8,818 | 45.6 | 2,405 (27.3) | 3,227 (36.6) | 2,606 (29.5) | 580 (6.6) |
| Suburban | 8,818 | 14.8 | 960 (33.4) | 973 (33.9) | 735 (25.6) | 205 (7.1) |
| Rural area | 7,628 | 39.5 | 2,844 (37.3) | 2,520 (33) | 1,696 (22.2) | 568 (7.4) |
| **Health Status** |  |  |  |  |  |  |
| Excellent | 2,619 | 13.6 | 1,122 (42.8) | 923 (35.2) | 417 (15.9) | 157 (6) |
| Good | 14,268 | 73.8 | 4,671 (32.7) | 5,068 (35.5) | 3,560 (24.9) | 969 (6.8) |
| Fair | 2,164 | 11.2 | 362 (16.7) | 657 (30.4) | 949 (43.8) | 196 (9.1) |
| Poor | 184 | 1 | 22 (12) | 51 (27.7) | 95 (51.6) | 16 (8.7) |
| Missing | 84 | 0.4 | 32 (38.1) | 26 (25) | 16 (19) | 15 (17.9) |
| **Depression status** |  |  |  |  |  |  |
| Normal | 12,361 | 68.8 | 5334 (38.5) | 5073 (37.3) | 2596 (19.3) | 642 (4.9) |
| Mild Depression | 2,646 | 14.7 | 490 (17.5) | 944 (33.7) | 1,212 (43.3) | 154 (5.5) |
| Moderate Depression | 754 | 4.2 | 66 (9.2) | 208 (27.5) | 480 (63.3) | 50 (6.2) |
| Severe Depression | 286 | 1.6 | 11 (3.6) | 50 (16.6) | 225 (74.5) | 16 (5.3) |
| Missing | 1,919 | 10.7 | 630 (26.1) | 673 (27.9) | 616 (25.6) | 491 (20.4) |

“All p-values for chi-square were <0.05.”

**Supplementary Table 2. The summary of the association between each of the oral frailty related exposures and social frailty without multiple imputation (complete case analysis)*.**

|  | **Crude Model** | | | **Adjusted Model** | | |
| --- | --- | --- | --- | --- | --- | --- |
| **Variables** | **OR** | **95%** | **CI** | **OR** | **95%** | **CI** |
| **Oral Frailty** (N= 14,257) |  |  |  |  |  |  |
| Robust (ref) | 1 |  |  | 1 | . | . |
| Pre oral frail | 1.2 | 1.17 | 1.33 | 1.2 | 1.09 | 1.27 |
| Oral Frailty | 1.8 | 1.71 | 1.95 | 1.4 | 1.25 | 1.47 |
| **Oral Frailty Score** (N= 14,257) |  |  |  |  |  |  |
| 0 | 1 |  |  | 1 |  |  |
| 1 | 1.25 | 1.17 | 1.33 | 1.18 | 1.09 | 1.27 |
| 2 | 1.61 | 1.49 | 1.73 | 1.30 | 1.19 | 1.43 |
| 3 | 2.02 | 1.82 | 2.24 | 1.39 | 1.23 | 1.58 |
| 4 | 2.62 | 2.23 | 3.08 | 1.53 | 1.25 | 1.86 |
| 5 | 4.91 | 3.18 | 7.57 | 2.33 | 1.28 | 4.23 |
| **Number of teeth** (N= 14,257) |  |  |  |  |  |  |
| ≥20 | 1 |  |  | 1 |  |  |
| <20 | 1.21 | 1.14 | 1.28 | 1.09 | 1.02 | 1.17 |
| **Chewing Difficulty** (N= 14,232) |  |  |  |  |  |  |
| No | 1 |  |  | 1 |  |  |
| Yes | 1.61 | 1.52 | 1.71 | 1.20 | 1.12 | 1.29 |
| **Swallowing Difficulty** (N= 14,226) |  |  |  |  |  |  |
| No | 1 |  |  | 1 |  |  |
| Yes | 1.45 | 1.36 | 1.55 | 1.25 | 1.15 | 1.34 |
| **Dry mouth** (N= 14,177) |  |  |  |  |  |  |
| No | 1 |  |  | 1 |  |  |
| Yes | 1.7 | 1.59 | 1.82 | 1.31 | 1.21 | 1.42 |
| **Speaking Difficulty** (N= 14,257) |  |  |  |  |  |  |
| No | 1 |  |  |  |  |  |
|  | 1.36 | 1.19 | 1.56 | 0.98 | 0.83 | 1.16 |
| **Oral Functions (OF)** (N= 14,150) |  |  |  |  |  |  |
| 0 diminished OF | 1 |  |  | 1 |  |  |
| 1 diminished OF | 1.38 | 1.29 | 1.47 | 1.15 | 1.07 | 1.24 |
| 2 diminished OF | 1.88 | 1.73 | 2.04 | 1.38 | 1.25 | 1.53 |
| 3 diminished OF | 2.40 | 2.11 | 2.73 | 1.45 | 1.24 | 1.69 |
| 4 diminished OF | 4.63 | 3.13 | 6.84 | 2.33 | 1.35 | 4.01 |

*Each oral frailty related exposure was included in a separate model twice (a crude and an adjusted model), and the results were summarized in the above table.

All p-values <0.005

Abbreviations: AOR: adjusted odds ratio, CI: confidence interval

Adjusted for: age, gender, education level, resident area, equivalized income, health status, and depression status

**Supplementary Table 3. Comparison of adjusted odds ratios (AORs) for the association between oral frailty as a categorical variable and social frailty after multiple imputation with covariates included (N = 19,319).**

| **Variables** | **AOR** | **95%** | **CI** |
| --- | --- | --- | --- |
| **Oral Frailty** |  |  |  |
| Robust (ref) | 1 |  |  |
| Pre oral frail | 1.13 | 1.05 | 1.21 |
| Oral Frailty | 1.35 | 1.26 | 1.45 |
| **Sex** |  |  |  |
| Male (ref) | 1 |  |  |
| Female | 0.81 | 0.77 | 0.86 |
| **Age** |  |  |  |
| 65-69 years (ref) | 1 |  |  |
| 70-74 years | 0.86 | 0.79 | 0.92 |
| 75-79 years | 0.81 | 0.74 | 0.88 |
| 80-84 years | 1.01 | 0.91 | 1.10 |
| 85 or more | 1.59 | 1.40 | 1.8 |
| **Education** |  |  |  |
| Less than 6 years | 1.04 | 0.64 | 1.68 |
| 6 to 9 years | 0.99 | 0.90 | 1.08 |
| 10 to 12 years | .974 | 0.91 | 1.04 |
| 13 years -more (ref) | 1 |  |  |
| **Equivalized income** |  |  |  |
| <2,000,000 Yen/Year | 1.58 | 1.43 | 1.74 |
| 2,000,000 - 2,999,999 Yen/Year | 1.27 | 1.15 | 1.42 |
| 3,000,000 - 3,999,999 Yen/Year | 1.03 | 0.93 | 1.14 |
| > 4,000,000 Yen/Year (ref) | 1 |  |  |
| **Resident area** |  |  |  |
| urban (ref) | 1 |  |  |
| suburb | 0.69 | 0.64 | 0.76 |
| rural | 0.53 | 0.50 | 0.57 |
| **Health Status** |  |  |  |
| Excellent (ref) | 1 |  |  |
| Good | 1.34 | 1.23 | 1.45 |
| Fair | 2.26 | 2.00 | 2.55 |
| Poor | 2.68 | 1.92 | 3.76 |
| **Depression** |  |  |  |
| Normal (ref) | 1 |  |  |
| Mild Depression | 2.84 | 2.62 | 3.09 |
| Moderate Depression | 5.55 | 4.74 | 6.50 |
| Severe Depression | 10.25 | 7.76 | 13.55 |

Abbreviations: AOR: adjusted odds ratio, CI: confidence interval

The data was imputed 20 times, and the analysis was conducted using ordered logistic regression

**Supplementary Table 4. The summary findings for the association of the number of teeth with different cut-off points and social frailty (N= 14,257)**

|  | **Crude Model** | | | **Adjusted Model** | | |
| --- | --- | --- | --- | --- | --- | --- |
| **Variables** | **OR** | **95%** | **CI** | **OR** | **95%** | **CI** |
| **Number of teeth** |  |  |  |  |  |  |
| ≥5 | 1 |  |  | 1 |  |  |
| <5 | 1.32 | 1.21 | 1.44 | 1.17 | 1.05 | 1.30 |
| **Number of teeth** |  |  |  |  |  |  |
| Non-edentulus | 1 |  |  | 1 |  |  |
| Edentulus | 1.33 | 1.18 | 1.49 | 1.21 | 1.05 | 1.40 |
